# Enhancing water productivity and yield of spring wheat through irrigation and planting density optimization under shallow-buried drip irrigation

**DOI:** 10.3389/fpls.2026.1875868

**Published:** 2026-07-20

**Authors:** Jie Zhou, Xiaoyuan Bao, Congcong Guo, Hong Fan, Bo Jing, Yali Sun, Wei He, Fuyang Cui, Xiaoya Gao, Lili Yang, Cai Zhao

**Affiliations:** 1 State Key Laboratory of Aridland Crop Science, College of Agronomy, Gansu Agricultural University, Lanzhou, China; 2 Seed Industry Research Institute of Gansu Provincial University, Lanzhou, China

**Keywords:** grain filling, planting density, shallow-buried drip irrigation, source–sink coordination, spring wheat, water productivity

## Abstract

**Introduction:**

Improving spring wheat production in oasis irrigation districts requires management strategies that reconcile high grain yield with efficient irrigation water use.

**Methods:**

A two-year field experiment was conducted in the Hexi Oasis, Northwest China, during 2024–2025 to evaluate the coupled effects of three planting densities (PD1, 10.0 million seeds ha^–1^; PD2, 10.8 million seeds ha^–1^; and PD3, 11.6 million seeds ha–1) and three irrigation levels (I1, 360 mm; I2, 315 mm; and I3, 270 mm) on grain yield, soil water use, crop water productivity (WP_C_), irrigation water productivity (WP_I_), dry matter translocation, and grain filling under shallow-buried drip irrigation.

**Results:**

Grain yield was consistently maximized under PD3I1, reaching 8455 and 8796 kg ha–1 in 2024 and 2025, respectively, whereas PD2I2 achieved a more favorable balance between yield and water productivity. Compared with I1, I2 reduced grain yield by only 5.3% and 6.0% in 2024 and 2025, respectively, but increased WP_C_ and WP_I_ by 5.5% on average. Across planting densities, PD2 increased WPC and WPI by 6.4% and 7.1% relative to PD1, respectively. Soil water profiles showed that reduced irrigation increased reliance on stored water in the 40–100 cm profile, particularly under high density. Grain filling followed a sigmoidal trajectory, with irrigation primarily regulating grain-filling rate, grain weight, and active filling duration, while excessive density restricted individual grain filling under water deficit.

**Discussion:**

Overall, moderate planting density combined with moderate irrigation provided a practical strategy for sustaining yield while enhancing irrigation water productivity in arid oasis wheat systems, with the benefits being primarily attributable to improved management coordination rather than to novel physiological mechanisms.

## 1 Introduction

Water scarcity is increasingly constraining irrigated crop production in dryland regions, making it necessary to produce more grain with less water and lower environmental cost. In wheat-based systems, irrigation usually increases grain yield, but the gain in water productivity is often limited because irrigation also raises crop water consumption, creating a persistent yield-water productivity trade-off (Yang et al., 2025). This issue is particularly relevant in oasis irrigation areas of Northwest China, where spring wheat production depends heavily on irrigation scheduling under restricted water resources (Li and Wang, 2021). Meta-analyses have shown that deficit irrigation can improve wheat water-use efficiency or water productivity, but its effects on yield vary substantially with stress timing and local management conditions (Yu et al., 2020).

Drip irrigation and subsurface drip irrigation are increasingly recognized as effective water-saving technologies because they can reduce non-productive soil evaporation, improve the soil water and thermal environment in the root zone, and enhance irrigation water productivity when properly managed (Wang et al., 2022). Field studies in Northwest China have shown that drip irrigation can alter evapotranspiration and its components relative to conventional surface irrigation, and can improve spring wheat yield and crop water productivity while reducing leaf area, indicating that canopy development and water consumption patterns are key determinants of productivity gains (Yang et al., 2023; Yang et al., 2020a). However, irrigation management alone may not be sufficient to optimize both yield and water productivity.

Planting density is another controllable management lever that regulates canopy structure, radiation interception, and intra population competition, and it often interacts with irrigation in determining yield and water productivity (Zhang et al., 2023). Under limited irrigation, increasing seeding density has been shown to improve winter wheat yield and water productivity by constructing a more favorable population architecture, including enhanced deep soil root length density (Gao et al., 2021). Recent evidence further indicates that irrigation and planting density should be optimized jointly, as their interaction can influence canopy traits, soil water use, evapotranspiration, and final water productivity (Dai et al., 2022).

Yield formation in wheat is governed by coordinated carbon supply, sink activity, and assimilate transport during grain filling (Fang et al., 2024). Irrigation at critical stages can alter dry matter accumulation and remobilization, thereby affecting grain filling and yield stability (Moradi et al., 2022). Under drip-based water and nutrient management, optimized irrigation and fertilization have been shown to improve post-anthesis dry matter accumulation and translocation, linking management directly to grain filling and final yield (Yan et al., 2022). In addition, grain filling and drought adaptation are associated with hormonal signaling and oxidative and osmotic protection. Recent studies have shown that moderate post-anthesis water limitation may involve cytokinin-mediated source-to-sink remobilization, while abscisic acid and brassinosteroids are also closely related to drought response and grain development in wheat (Luo et al., 2021; Ru et al., 2024; Zolkiewicz and Gruszka, 2025).

Despite these advances, many previous studies have focused either on agronomic outcomes such as yield and water productivity or on individual physiological traits, whereas fewer studies have integrated irrigationcalE.DAT coupling with soil water use, grain filling, dry matter translocation, and related physiological responses in spring wheat under oasis irrigation conditions. Therefore, this study evaluated spring wheat under combined planting density and deficit irrigation treatments in the Hexi Oasis of Northwest China. Overall, moderate planting density combined with moderate irrigation improved the coordination between canopy demand, soil water supply, and grain filling, providing a practical strategy for sustaining yield while enhancing irrigation water productivity in arid oasis wheat systems.

## 2 Materials and methods

### 2.1 Experimental site

Field experiments were carried out during 2024–2025 at the Yongchang Experimental Station of Gansu Agricultural University, Jinchang City, Gansu Province, China (38°32’ N, 102°02’ E; elevation, 1850 m). The station is situated at the eastern end of the Hexi Corridor, a typical single-cropping area where limited thermal resources restrict double cropping. The region has a temperate continental arid climate, with a frost-free period of 134 days, a mean annual temperature of 4.8°C, accumulated temperatures of 3510.4°C above 0°C and 2011.2°C above 10°C, annual reference evapotranspiration of 2000.6 mm, and 2884.2 h of annual sunshine. Wheat is the dominant crop and is mainly produced under continuous monoculture.

**Figure 1:**
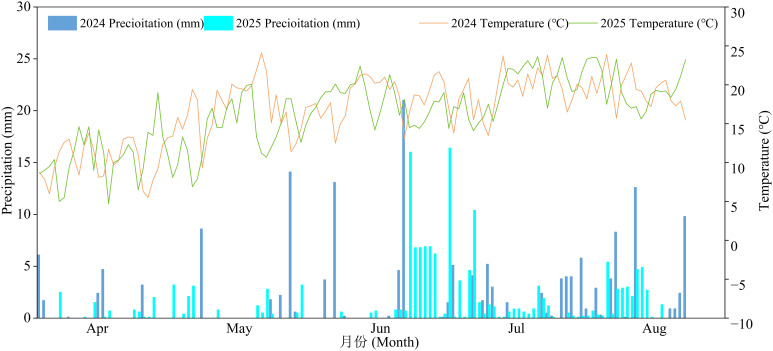
Climatic conditions during the 2024 and 2025 growing seasons at the experimental.

The soil is classified as irrigated desert soil. In the 0–30 cm layer, soil bulk density was 1.36 g cm^-3^, organic matter content was 16.3 g kg^-1^, total nitrogen was 0.89 g kg^-1^, and available phosphorus was 20.4 mg kg^-1^. Meteorological conditions during the 2024 and 2025 growing seasons are shown in **Figure 1**; total precipitation was 174.2 mm in 2024 and 163.9 mm in 2025.

### 2.2 Experimental design and field management

**Figure 2:**
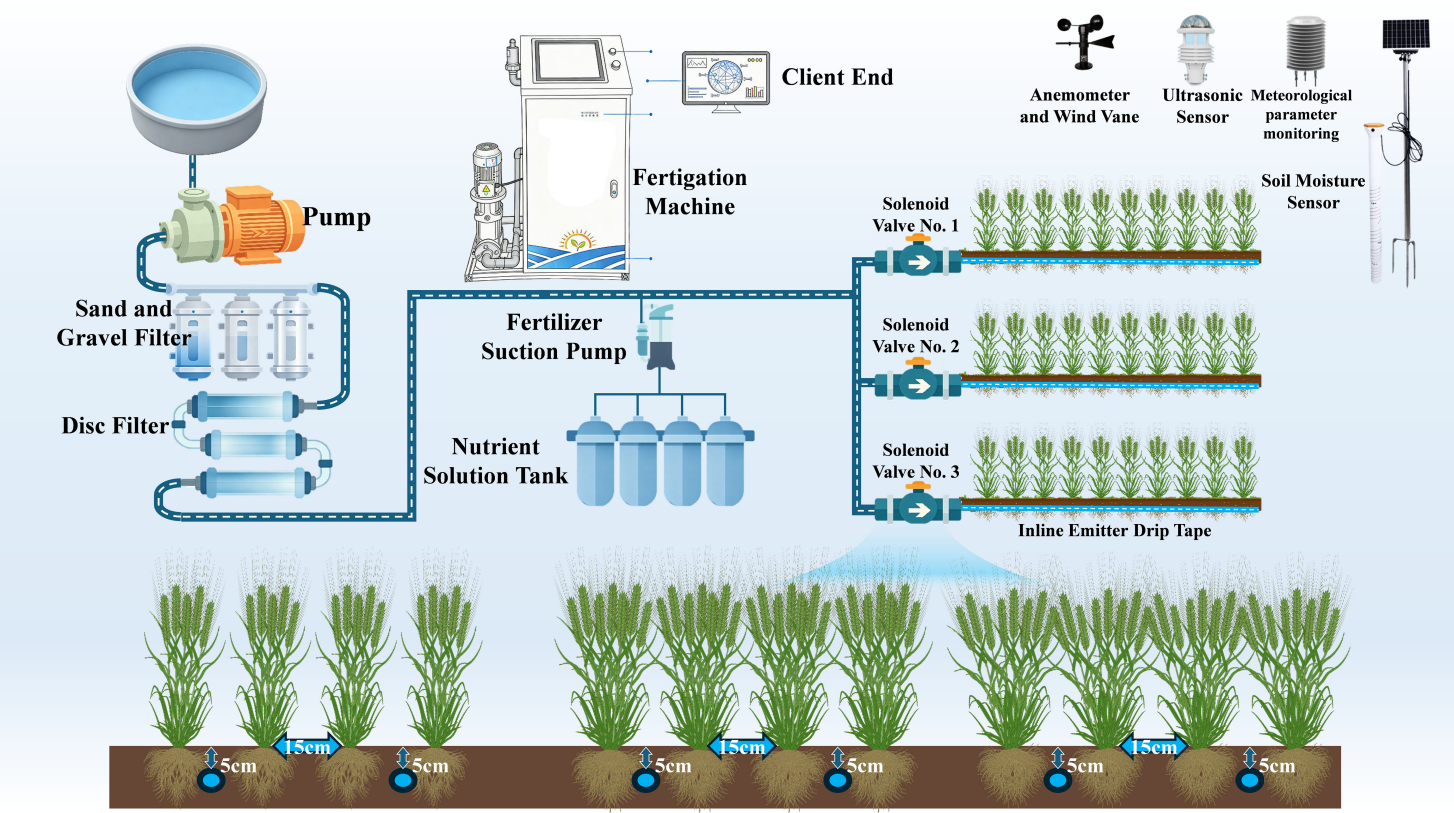
Schematic diagram of fertigation for wheat with shallow buried drip irrigation.

A two-factor split-plot design was employed, with seeding density assigned to main plots and irrigation level to subplots. Three seeding densities were established: PD1 (10.0 million grains ha^–1^), PD2 (10.8 million grains ha^–1^), and PD3 (11.6 million grains ha^–1^). Each density was combined with three irrigation levels: I1 (360 mm), I2 (315 mm), and I3 (270 mm). This arrangement resulted in nine treatment combinations, each replicated three times, for a total of 27 plots. Each plot was 7.5 m long and 4.8 m wide (36.0 m²). Row spacing was 15 cm, and the spring wheat cultivar used was Longchun 30 (**Figure 2**).

Fertilization was standardized across treatments, with 180 kg N ha^–1^ and 90 kg P_2_O_5_ ha^–1^ applied. The fertilizers included urea (46% N), single superphosphate (12% P_2_O_5_), and diammonium phosphate (18% N, 46% P_2_O_5_). Irrigation timing and the corresponding application amounts are provided in **Table 1**. The experiment was conducted over two consecutive growing seasons (2024–2025), completing two full crop cycles. Grain yield was measured at harvest each year, while physiological traits were assessed during the 2024 and 2025 growing seasons; soil volumetric water content was monitored over the same period. Pest, disease, and weed control followed local standard management practices. Spring wheat was sown on 7 April and harvested on 16 August in both 2024 and 2025.

**Table 1:** Irrigation schedule for spring wheat.

Growth stage	I1 (mm)	I2 (mm)	I3 (mm)
Seedling Emergence	25.0	22.5	20.0
Tillering Stage	45.0	40.0	35.0
Jointing Stage	75.0	65.0	55.0
Booting Stage	85.0	75.0	65.0
Heading–Flowering Stage	80.0	70.0	60.0
Grain Filling Stage	50.0	42.5	35.0
Total Irrigation Amount	360.0	315.0	270.0

### 2.3 Methods for field investigation

#### 2.3.1 Soil water storage and water productivity

Soil volumetric water content (SWC) was measured at 15-day intervals throughout the growing season using a handheld time-domain reflectometry instrument (Trime Pico 64 Portable Soil Moisture Meter, TDR, EMIKO, GmbH, Bochum, Germany). Measurements were taken from the 0–120 cm soil profile at 20 cm intervals. At each sampling date, readings were collected from representative locations within each plot and averaged to obtain the SWC value for each soil layer. Soil water storage (SWS, mm) in the 0–120 cm profile was calculated based on the volumetric water content and the thickness of each soil layer. Soil water content was calculated as:

(1) $$\begin{array}{c} SWS=\overset{n}{\underset{i=1}{\sum }}\theta_{i}\times H_{i} \end{array}$$

Where ${\text{θ}}_{\text{i}}\;$ is the volumetric soil water content of the ith soil layer (%), ${\text{H}}_{\text{i}}\;$ is the thickness of the ith soil layer (mm), and n is the number of soil layers.

Crop evapotranspiration (ET) during the wheat growth period was calculated using the soil water balance equation:

(2) $$\begin{array}{c} ET=I+P-RF-DD\pm \text{Δ}SWS \end{array}$$

where I (mm) is the irrigation amount, P (mm) is the rainfall amount, RF (mm) is the surface runoff, DD (mm) is the deep drainage, and ΔSWS (mm) is the change in soil water storage in the 0–120 cm soil layer between the start and end of the experiment. Due to the flat terrain of the experimental site and limited water percolation in the 0–120 cm soil layer, RF and DD were neglected.

Crop water productivity (WP_C_) and irrigation water productivity (WP_I_) were calculated as follows:

(3) $$\begin{array}{c} {\text{WP}}_{\text{C}}=\frac{\text{GY}}{\text{ET}} \end{array}$$

(4) $$\begin{array}{c} {\text{WP}}_{\text{I}}=\frac{\text{GY}}{\text{I}} \end{array}$$

where GY is the grain yield of a certain treatment (kg ha^–1^), ET (mm) is the actual evapotranspiration during the growth period, and I is the total irrigation amount (mm). All data were finally converted to kg m^-3^ using a conversion factor of 0.1 (1 kg ha^-1^ mm^-1^ = 0.1 kg m^-3^).

#### 2.3.2 Dry matter accumulation and translocation

The aboveground parts of all plants from 0.2 m^2^ (avoid borders) in each plot were sampled at anthesis and maturity stages. These plants were then oven–dried at 75°C until consistent weight was achieved. The biomass was subsequently quantified. The parameters related to dry matter accumulation (DM) and remobilization within the wheat plant were determined by following equations:

(5) $$\begin{array}{c} DM_{maturity,veg}=DM_{anthesis}-GY \end{array}$$

(6) $$\begin{array}{c} TA_{pre}=DM_{maturity,veg}-DM_{anthesis} \end{array}$$

(7) $$\begin{array}{c} TE_{pre}=\frac{TA_{pre}}{DM_{anthesis}}\times 100\% \end{array}$$

(8) $$\begin{array}{c} CR_{pre}=\frac{TA_{pre}}{GY}\times 100\% \end{array}$$

(9) $$\begin{array}{c} TA_{post}=GY-TA_{pre} \end{array}$$

(10) $$\begin{array}{c} CR_{post}=\frac{TA_{post}}{GY}\times 100\% \end{array}$$

where TA_pre_ is pre-anthesis dry matter translocation (kg ha^–1^); DM_anthesis_ is dry matter at anthesis (kg ha^–1^); DM_maturity, veg_ is dry matter of the vegetative organs at maturity (kg ha^–1^); TA_post_ is post-anthesis dry matter accumulation (kg ha^–1^); GY is grain dry matter at maturity (kg ha^–1^); TE_pre_ is dry matter translocation efficiency (%); CR_pre_ is contribution of pre-anthesis DM to grain yield (%); CR_post_ is contribution of post-anthesis DM to grain yield (%).

#### 2.3.3 Grain filling rate

Two hundred spikes that reached anthesis on the same day were selected and tagged in each plot. For each treatment, twenty tagged spikes were sampled at 6-day intervals from anthesis to maturity. After sampling, spikes were heated at 105°C for 10 min, then oven-dried at 70°C to constant weight and weighed. Grain filling was described using a logistic function as follows:

(11) $$\begin{array}{c} W=\frac{\text{a}}{\left(1+{\text{be}}^{-\text{ct}}\right)} \end{array}$$

where W (g) is the 1000–grain dry weight at time; a (g) is the theoretical final 1000–grain dry weight; t (d) is time after anthesis; and b and c are the coefficients determined by regression. Based on the fitted parameters, the time required to reach maximum grain–filling rate (T_max_); the duration of grain filling (T), the grain weight at maximum grain filling rate (W_max_); maximum grain filling rate (V_max_); mean grain filling rate (V_mean_); and active grain filling period (D) were calculated using the following equations, according to the method proposed by (He et al., 2020):

(12) $$\begin{array}{c} {\text{T}}_{\text{max}}=\frac{\text{lnb}}{\text{c}} \end{array}$$

(13) $$\begin{array}{c} T=\frac{\left(\text{lnb}+4.59512\right)}{\text{c}} \end{array}$$

(14) $$\begin{array}{c} {\text{W}}_{\text{max}}=\frac{\text{a}}{2} \end{array}$$

(15) $$\begin{array}{c} {\text{V}}_{\text{max}}=c\times {\text{W}}_{\text{max}}\times (1-\frac{{\text{W}}_{\text{max}}}{\text{a}}) \end{array}$$

(16) $$\begin{array}{c} {\text{V}}_{\text{mean}}=\frac{a}{\text{T}} \end{array}$$

(17) $$\begin{array}{c} D=\frac{5}{\text{c}} \end{array}$$

#### 2.3.4 Leaf area index

Leaf area index (LAI) was measured with an LAI-2200 Plant Canopy Analyzer (LI-COR, Lincoln, NE, USA) under diffuse light conditions to avoid direct sunlight interference. Measurements were taken on clear days between 09:00 and 11:30 h by recording readings above and below the canopy. For each plot, ten readings were collected and averaged to obtain a single LAI value.

#### 2.3.5 Photosynthetic characteristics and gas exchange parameters

Flag leaf gas exchange parameters, including net photosynthetic rate (Pn), transpiration rate (Tr), stomatal conductance (Gs) and intracellular CO_2_ concentration (Ci), were measured using Li-6800 portable photosynthetic system (LI-COR Biosciences, USA). Measurements were conducted at the jointing, booting, and anthesis stages, as well as during grain filling (20 days after anthesis), between 09:00–12:00 h, with photosynthetically active radiation set as 1000 μ mol m^–2^ s^–1^ and reference CO_2_ concentration of 400 μ mol m^–2^ s^–1^. Three uniformly sized flag leaves per plot were measured.

#### 2.3.6 Physiological indicators and hormone contents

Flag leaves were sampled at mid-grain filling, frozen in liquid nitrogen, and stored at -80 °C. Samples (0.5 g) were homogenized in 5 mL of ice-cold phosphate buffer (pH 7.8) and centrifuged at 12, 000 × g for 20 min at 4 °C. SOD was determined by the NBT photoreduction method (560 nm), POD by the guaiacol method (470 nm), and PRO by the ninhydrin method (520 nm) (Bao et al., 2023). Enzyme activities were expressed as U g^-1^ FW and PRO as μg g^-1^ FW. Three analytical replicates were performed per sample, with a QC sample in each batch (Bao et al., 2023).

Frozen flag leaf samples (1.0 g) were extracted with 10 mL of 80% methanol (containing 0.1% BHT) at 4 °C for 12 h, centrifuged at 10, 000 × g for 15 min, and the supernatant was purified using a C18 cartridge. The eluate was dried and reconstituted in 1 mL methanol. ZR, BR, and ABA were quantified by UPLC-MS/MS, the mainstream method for trace endogenous phytohormone detection, on a C18 column (4.6 × 250 mm, 5 μm) with methanol–0.1% acetic acid at 1.0 mL min^-1^ under MRM mode. External standard curves (0.1–20 μg mL^-1^, R² > 0.99) were used. Concentrations were expressed as ng g^-1^ FW (Xin et al., 2020).

#### 2.3.7 Grain yield and yield components

At physiological maturity, three 1 m^2^ quadrats were established in the central area of each plot to minimize border effects. Plants within each quadrat were manually harvested and oven-dried at 70 °C to constant mass. Panicle number per unit area was counted directly. Twenty panicles were then randomly selected to determine the number of filled grains per panicle. Thousand-grain weight (TGW) was measured by weighing three subsamples of 1000 well-filled grains using an electronic balance (0.001 g precision), and the mean value was used for analysis. Grain yield (GY) was calculated from the dry grain mass harvested from the quadrats and converted to kg ha^-1^. Harvest index (HI) was calculated as the ratio of grain yield to dry matter accumulation at maturity.

### 2.4 Statistical analysis

Data were analyzed using IBM SPSS Statistics 27.0 (IBM Corp., Armonk, NY, USA). Analysis of variance appropriate for a split-plot design was applied, with planting density as the main-plot factor and irrigation level as the subplot factor. Prior to analysis, normality and homogeneity of variance were tested using the Shapiro–Wilk and Levene’s tests, respectively. Mean comparisons were performed using the least significant difference (LSD) test at *P* < 0.05. Figures were prepared using Origin 2021 (OriginLab, Northampton, MA, USA), and structural equation modeling (SEM) was conducted using AMOS 26.0 (IBM Corp., Armonk, NY, USA).

## 3 Results

### 3.1 Yield and yield components

Planting density (PD) and irrigation level (I) significantly affected spike number, grain yield (GY) and harvest index (HI), and their interaction significantly affected GY (*P*<0.05, **Table 2**). Compared with I1, mean GY under I2 and I3 decreased by 5.3% and 22.2% in 2024, and by 6.0% and 22.4% in 2025, respectively. The yield reduction under I3 was accompanied by significant declines in spike number, grains per spike, and thousand-grain weight (TGW). At the same irrigation level, grains per spike and TGW generally decreased with increasing planting density. In 2024, GY increased with planting density under I1, with PD3 reaching 8455 kg ha^-1^, 11.7% higher than PD1. Under I2, GY showed a unimodal response and peaked at PD2 (8101 kg ha^–1^), 11.8% higher than PD1, under I3, grain yield did not decrease with density, and PD3 was 4.1% higher than PD1.

**Table 2:** Impact of various planting densities and irrigation treatments on plant, yield and its components of spring wheat in 2024 and 2025.

Year	Planting density	Irrigation	Spike number (×10^4^ ha^–1^)	Grain number per spike	TGW (g)	Grain yield (kg ha^–1^)	Harvest index
2024	PD1	I1	561.0 de	41.6 a	41.7 a	7565.0 c	0.399 d
		I2	519.0 e	40.2 ab	38.2 bc	7244.6 c	0.458 a
		I3	519.7 e	38.2 c	32.5 d	6090.9 d	0.416 bcd
	PD2	I1	617.6 bc	39.5 bc	40.6 ab	8152.8 ab	0.444 abc
		I2	604.9 bcd	38.6 bc	38.6 abc	8101.2 b	0.437 abc
		I3	569.9 cde	35.6 d	32.6 d	6379.4 d	0.44 abc
	PD3	I1	693.3 a	38.5 bc	40.1 ab	8454.9 a	0.413 abc
		I2	614.6 bc	38.0 c	36.0 c	7542.7 c	0.435 cd
		I3	625.5 b	35.2 d	29.9 d	6339.6 d	0.448 ab
2025	PD1	I1	543.1 bc	39.9 a	42.0 a	7655.7 c	0.412 d
		I2	519.8 cd	37.8 abc	41.5 a	7167.9 d	0.396 d
		I3	505.5 d	37.4 abc	38.0 cd	6385.7 e	0.392 d
	PD2	I1	576.0 b	40.4 a	41.5 a	8291.4 b	0.444 ab
		I2	573.0 b	37.3 abc	40.8 ab	8195.3 b	0.453 ab
		I3	548.5 bc	37.2 abc	37.7 d	6601.3 e	0.400 d
	PD3	I1	634.3 a	39.1 ab	39.4 bc	8795.5 a	0.456 a
		I2	636.6 a	35.3 c	39.4 bc	7898.5 bc	0.431 bc
		I3	575.7 b	35.9 bc	32.4 e	6206.5 e	0.369 e
Planting density (PD)			**	ns	**	**	*
Irrigation (I)			*	**	ns	**	*
PD × I			ns	ns	ns	**	ns

TGW, Thousand-grain weight. Values followed by the same lowercase letter within a column for a given year are not significantly different at P < 0.05 according to the LSD test. * and ** indicate significant effects at the 0.05 and 0.01 probability levels, respectively; ns indicates not significant.

Across both seasons, the highest GY was consistently obtained under PD3I1, reaching 8455 kg ha^-1^ and 8796 kg ha^-1^ in 2024 and 2025. Relative to PD3I1, GY under PD2I1 and PD2I2 decreased by 4.7% and 5.5%, respectively, whereas other treatments showed larger reductions ranging from 10.5% to 27.7%. In contrast, HI was relatively higher under moderate density treatments, with PD2I1 and PD2I2 achieving two-year averages of 0.444 and 0.445, respectively.

### 3.2 Dry matter accumulation and translocation

In this study, pre-anthesis assimilate translocation amount (TA_pre_), translocation efficiency (TE_pre_), contribution of pre-anthesis reserves to grain (CR_pre_), contribution of post-anthesis assimilates to grain (CR_post_), and post-anthesis dry matter accumulation (TA_post_) were calculated using Equations 6–10, respectively. PD, I and their interaction significantly affected pre-anthesis assimilate translocation amount (TA_pre_), translocation efficiency (TE_pre_), the contribution of pre-anthesis reserves to grain (CR_pre_), and the contribution of post-anthesis assimilates to grain (CR_post_) (*P*<0.05; **Table 3**). Compared with I1, reducing irrigation increased TA_pre_, TE_pre_, and CR_pre_ but decreased CR_post_. In 2024, CR_pre_ under I3 reached 41.8%edednsi higher than the 19.5%–39.5% observed under I1; in 2025, CR_pre_ increased from 19.1%–31.3% under I1 to 38.3%–46.6% under I3. Correspondingly, CRpost decreased under reduced irrigation.

**Table 3:** Impact of various planting densities and irrigation treatments on plant, dry matter transport characteristics of spring wheat in 2024 and 2025.

Year	Planting density	Irrigation	Assimilation before anthesis			Assimilation after anthesis	
			TA_Pre_ (kg ha^–1^)	TE_Pre_ (%)	CR_Pre_ (%)	TA_Post_ (kg ha^–1^)	CR_Post_ (%)
2024	PD1	I1	1473.0 d	11.4 d	19.5 f	6092.8 a	80.5 a
		I2	2487.9 c	22.5 bc	34.3 e	4756.7 c	65.7 b
		I3	2545.3 c	22.9 bc	41.8 cd	3545.5 d	58.2 cd
	PD2	I1	3223.1 ab	24.0 bc	39.5 de	4929.7 c	60.5 bc
		I2	3094.1 ab	22.9 bc	38.2 de	5007.1 bc	61.8 bc
		I3	3370.2 ab	29.4 a	52.9 ab	3009.2 e	47.1 ef
	PD3	I1	2972.4 bc	19.9 c	35.2 de	5482.5 b	64.9 bc
		I2	3572.3 a	26.7 ab	47.3 bc	3970.4 d	52.7 de
		I3	3491.4 ab	30.9 a	55.0 a	2848.2 e	45.0 f
2025	PD1	I1	1467.7 d	11.8 d	19.1 f	9123.3 b	80.9 a
		I2	2485.0 bc	18.5 bc	34.6 cd	9652.9 b	65.4 cd
		I3	2682.3 abc	21.3 a	42.0 b	9068.1 b	58.0 e
	PD2	I1	2590.0 abc	20.0 abc	31.3 d	10881.4 a	68.7 c
		I2	2597.3 abc	20.8 ab	31.7 d	10792.6 a	68.3 c
		I3	2529.7 abc	20.3 abc	38.3 bc	9130.9 b	61.8 de
	PD3	I1	2303.7 c	18.0 c	26.2 e	11099.2 a	73.8 b
		I2	2942.7 a	22.0 a	37.2 c	10841.2 a	62.8 d
		I3	2889.7 ab	21.4 a	46.6a	9096.2 a	53.4 f
Planting density (PD)			**	**	**	ns	**
Irrigation (I)			**	**	**	ns	**
PD × I			**	*	**	ns	**

TA_pre_, pre-anthesis dry matter translocation (kg ha^–1^); DM_anthesis_, dry matter at anthesis (kg ha^–1^); TA_post_, post-anthesis dry matter accumulation (kg ha^–1^); TE_pre_, dry matter translocation efficiency (%); CR_pre_, contribution of pre-anthesis DM to grain yield (%); CR_post_, contribution of post-anthesis DM to grain yield (%). Values followed by the same lowercase letter within a column for a given year are not significantly different at P < 0.05 according to the LSD test. * and ** indicate significant effects at the 0.05 and 0.01 probability levels, respectively; ns indicates not significant.

**Figure 3:**
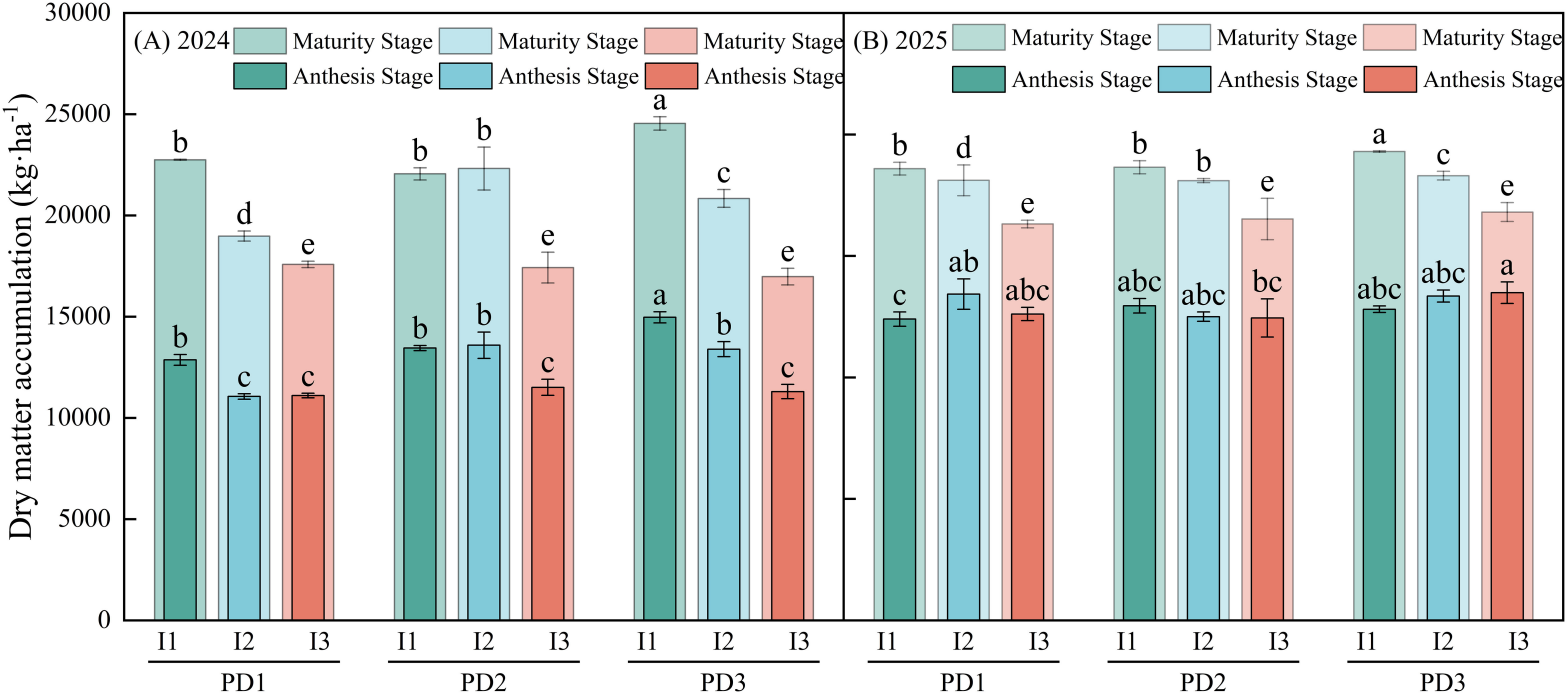
Effect of irrigation on dry matter accumulation of spring wheat under different irrigation treatments of 2024 **(a)** and 2025 **(b)**. The data are shown as the means ± SDs. Different letters indicate significant differences between different treatments (*P* < 0.05).

At the same irrigation level, higher density generally increased TA_pre_ and CR_pre_ but reduced CRpost. In 2024, TA_pre_ was highest under PD3I2 (3572.3 kg ha^-1^), while TE_pre_ and CR_pre_ peaked under PD3I3 (30.9% and 55.0%, respectively). The dry matter of vegetative organs at maturity (DMmaturity, veg) was calculated using Equation 5. In 2025, TA_post_ was highest under PD2I1 and PD3I1 (**Figure 3**). Across years, CR_post_ was generally higher than CR_pre_, showing that post-anthesis assimilation remained the dominant contributor to grain dry matter.

### 3.3 Crop phenology

**Figure 4:**
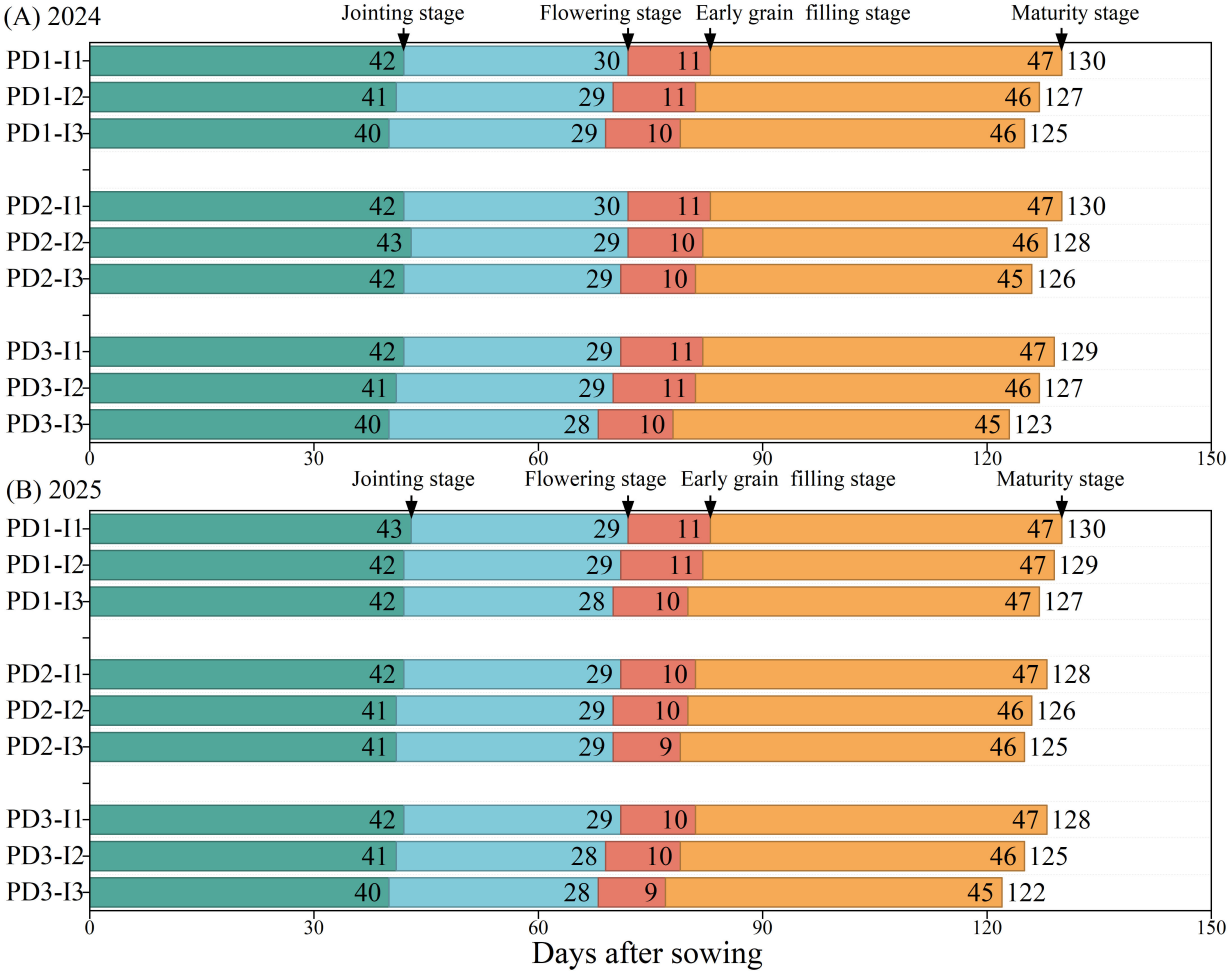
Effects of irrigation and planting density application rates on growth process of spring wheat in 2024 **(a)** and 2025 **(b)**.

PD and I affected spring wheat phenology consistently across the two years (**Figure 4**). At the same density, reducing irrigation from I1 to I3 advanced developmental stages and shortened the total growth duration. Under PD1, I3 shortened the growth period by 5 days in 2024 and 3 days in 2025 compared with I1. The early grain-filling stage was most sensitive to irrigation reduction, with its duration under I3 being 9.1% shorter than under I1 on average.

At the same irrigation level, increasing density from PD1 to PD3 also accelerated development. Under I1, PD3 shortened the total growth period by 1 day in 2024 and 2 days in 2025 compared with PD1. The shortest growth duration occurred under PD3I3, which was 7 and 8 days shorter than PD1I1 in 2024 and 2025, respectively.

### 3.4 Crop water productivity and water consumption characteristics

In this study, soil water storage variation (ΔSWS), evapotranspiration (ET), crop water productivity (WPC), and irrigation water productivity (WPI) were quantified using Equations 1–4. I and PD significantly affected the change in soil water storage (ΔSWS), evapotranspiration (ET), crop water productivity (WPC), and irrigation water productivity (WPI) (P < 0.05), while their interaction significantly affected ET and WPC (**Table 4**). Compared with I1, I2 and I3 significantly increased ΔSWS in both years, indicating greater crop dependence on stored soil water under reduced irrigation. From I1 to I3, ΔSWS increased from 13.4–16.1 mm to 18.1–25.3 mm in 2024, and from 11.9–22.4 mm to 17.2–25.0 mm in 2025. In contrast, ET decreased markedly with decreasing irrigation amount, ranging from 462.3 to 550.3 mm in 2024 and from 451.1 to 546.3 mm in 2025.

**Table 4:** Evaporation amount (ET), soil water consumption composition (ΔSWS), crop water productivity (WP_C_) and irrigation water productivity (WP_I_) under different irrigation treatments.

Year	Treatment	I (mm)	P (mm)	ΔSWS (mm)	ET (mm)	WP_C_ (kg m^-3^)	WP_I_ (kg m^-3^)
2024	PD1I1	360	174.2	13.9 e	548.1 a	1.38 de	2.10 e
	PD1I2	315	174.2	15.1 de	504.3 c	1.44 cd	2.30 cd
	PD1I3	270	174.2	18.1 cd	462.3 e	1.35 e	2.31 cd
	PD2I1	360	174.2	13.4 e	547.6 a	1.46 bc	2.23 d
	PD2I2	315	174.2	19.8 bc	509.0 b	1.53 ab	2.48 a
	PD2I3	270	174.2	25.3 a	469.5 d	1.39 de	2.42 ab
	PD3I1	360	174.2	16.1 de	550.3 a	1.54 a	2.35 bc
	PD3I2	315	174.2	22.3 ab	511.5 b	1.51 ab	2.46 ab
	PD3I3	270	174.2	22.3 ab	466.5 d	1.36 e	2.35 bc
2025	PD1I1	360	163.9	11.9 f	535.8 c	1.43 de	2.13 d
	PD1I2	315	163.9	17.8 d	496.7 e	1.44 d	2.28 c
	PD1I3	270	163.9	20.8 bc	454.7 g	1.4 de	2.37 bc
	PD2I1	360	163.9	14.7 e	538.6 b	1.54 bc	2.30 bc
	PD2I2	315	163.9	19.5 cd	498.4 de	1.64 a	2.59 a
	PD2I3	270	163.9	17.2 de	451.1 h	1.46 cd	2.44 b
	PD3I1	360	163.9	22.4 b	546.3 a	1.61 ab	2.44 b
	PD3I2	315	163.9	21.7 bc	500.6 d	1.64 a	2.60 a
	PD3I3	270	163.9	25.0 a	458.9 f	1.35 e	2.30 bc
Planting density (PD)		*			***	***	***
Irrigation (I)		***			***	***	***
PD×I		ns			***	***	ns

I, the total irrigation amount; P, is the rainfall amount; ΔSWS, the change in soil water storage in the 0–120 cm soil layer between the start and end of the experiment; ET, the actual evapotranspiration during the growth period; WP_C_, crop water productivity; WP_I_, irrigation water productivity. Values followed by the same lowercase letter within a column for a given year are not significantly different at P < 0.05 according to the LSD test. * and ** indicate significant effects at the 0.05 and 0.01 probability levels, respectively; ns indicates not significant.

WPC and WPI were both generally higher under I2 than under I1 and I3. Compared with I1 and I3, I2 increased WPC by 2.7% and 10.7% on average, respectively, and increased WPI by 8.6% and 3.7%, respectively. At the same irrigation level, WPC and WPI generally increased from PD1 to PD2 and then remained stable or slightly declined at PD3. Compared with PD1, PD2 increased WPC and WPI by 6.9% and 7.2% on average, respectively. PD2I2 and PD3I2 showed the highest overall water productivity, with WPC of 1.53 and 1.64 kg m^-3^ and WPI of 2.48 and 2.60 kg m^-3^ in 2024 and 2025, respectively. However, compared with PD3I2, PD2I2 achieved comparable WPC and WPI with lower ΔSWS and ET, indicating that moderate irrigation combined with moderate planting density provided a better balance among crop water use, soil water depletion, and irrigation water productivity.

### 3.5 LAI and gas exchange parameters

**Figure 5:**
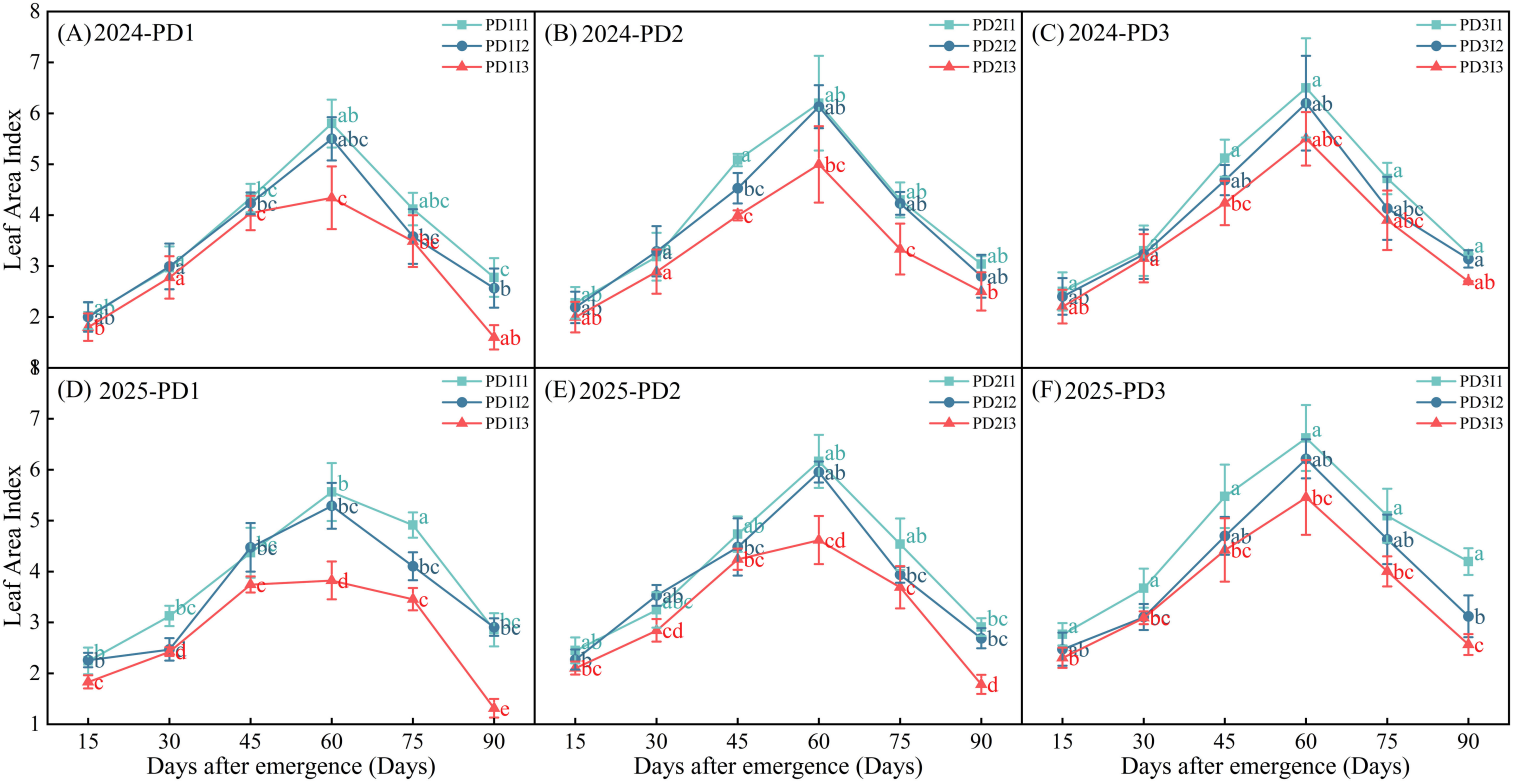
Effect of different irrigation and planting densities on the leaf area index of wheat in 2024 and 2025. **(a)** 2024-PD1; **(b)** 2024-PD2; **(c)** 2024-PD3; **(d)** 2025-PD1; **(e)** 2025-PD2; **(f)** 2025-PD3. The legends for each treatment group are placed at the top-right corner of each subplot. Data are presented as means ± SDs. Different lowercase letters denote significant differences among treatments (P < 0.05).

**Figure 6:**
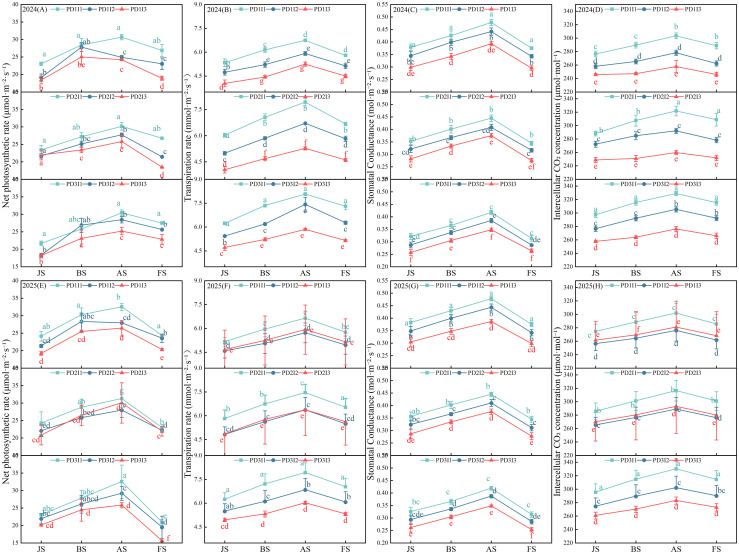
Effects of irrigation and planting densities on net photosynthetic rate (Pn), transpiration rate (Tr), stomatal conductance (Gs) and intracellular CO_2_ concentration (Ci) of wheat in 2024 and 2025. The data are shown as the means ± SDs. Different letters indicate significant differences between different treatments (*P* < 0.05).

PD and I significantly affected LAI and flag-leaf gas exchange parameters, whereas their interaction was significant only for Pn during grain filling (*P* < 0.05; **Figures 5**, **6**). Across treatments, LAI followed a unimodal pattern, peaking around anthesis and declining thereafter, indicating that this stage was critical for canopy development and maintenance.

At the same irrigation level, increasing planting density enhanced LAI, particularly during the mid-to-late growth stages. Under I2, LAI under PD3 was 2.7%, 7.3%, and 14.1% higher than under PD2 at 60, 75, and 90 DAS, respectively. At the same density, moderate irrigation reduction had limited effects on early canopy development but significantly influenced canopy persistence. Under PD3, LAI at 60 DAS under I2 was only 5.4% lower than under I1, whereas compared with I3, LAI under PD3I2 increased by 13.3%, 10.9%, and 19.0% at 60, 75, and 90 DAS, respectively.

Gas exchange parameters showed consistent responses across both seasons, with treatment differences most pronounced around anthesis. PD2I2 generally maintained higher Pn, Tr, Gs, and Ci. Around anthesis, Pn under PD2I2 was 2.5%–4.3% lower than under PD3I2 and 11.3% higher than under PD1I2 in 2024. Compared with I3, I2 increased Tr by 12.8%–27.1% and Gs by 8.7%–16.5% in 2024, and by 10.1%0.4, as in 2025 across densities. Ci was also higher under I2 than under I3, but not consistently in 2025, and I1 significantly differed from I3 in most cases.

### 3.6 Grain-filling dynamics and parameters

**Figure 7:**
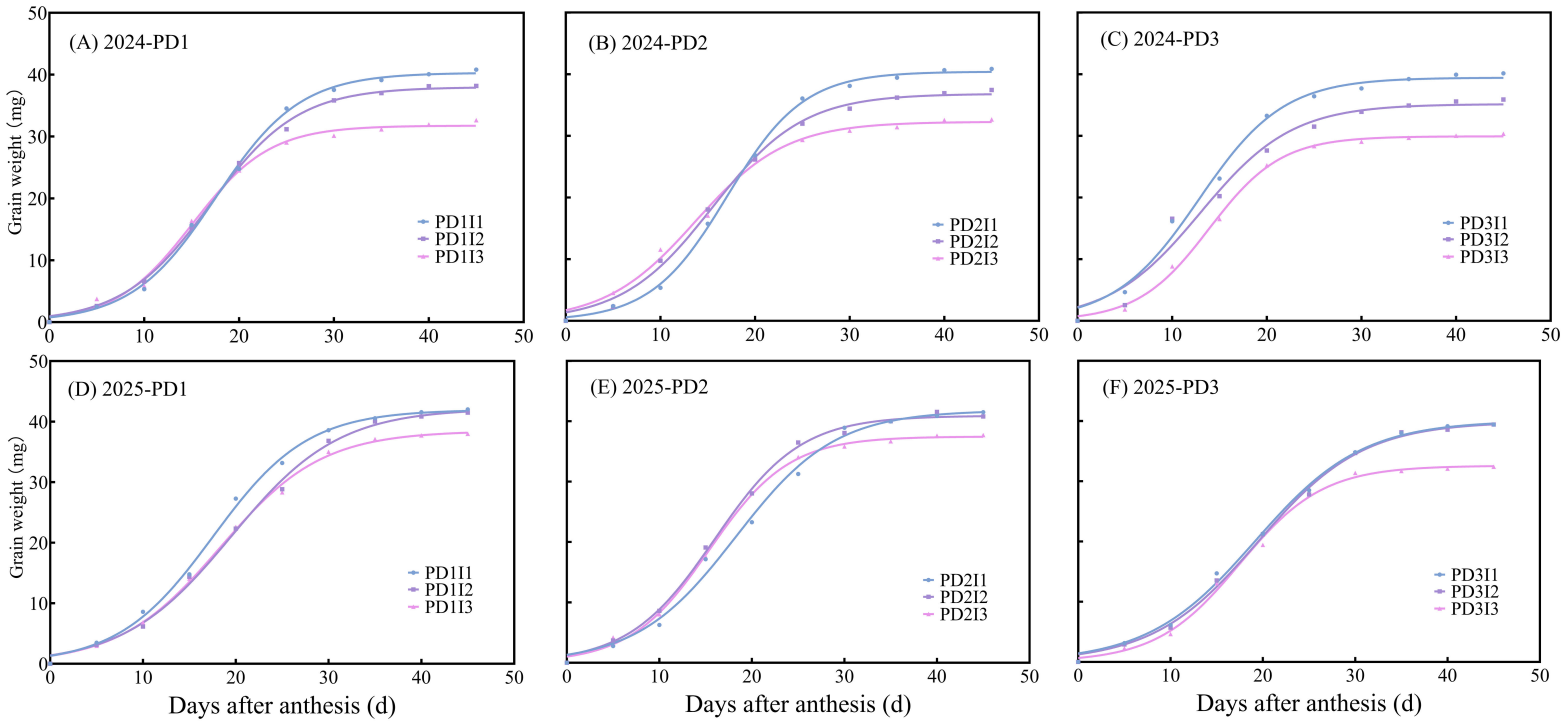
Changes in spring wheat grain weight after anthesis under different planting density and irrigation treatments in 2024 and 2025. Subplot definitions: **(a)** 2024-PD1; **(b)** 2024-PD2; **(c)** 2024-PD3; **(d)** 2025-PD1; **(e)** 2025-PD2; **(f)** 2025-PD3.

Grain weight followed a sigmoidal pattern and increased rapidly from 10 to 30 days after anthesis (DAA), with slightly higher final values in 2025 than in 2024 (**Figure 7**). Treatment differences were small during early grain filling but became more evident during the rapid-filling phase and at maturity. At the same irrigation level, increasing density reduced final grain weight, particularly under PD3. Across densities, grain weight declined as irrigation decreased, with the largest reduction occurring under I3.

The logistic model described the grain-filling process well across treatments (**Table 5**). In this study, the time required to reach the maximum grain-filling rate (Tmax), grain weight at the maximum grain-filling rate (Wmax), maximum grain-filling rate (Vmax), mean grain-filling rate (Vmean), and active grain-filling period (D) were quantified using Equations 11–17, respectively. Irrigation significantly affected maximum grain filling rate (V_max_), grain weight at maximum grain filling rate (W_max_)and active grain filling period (D), whereas planting density significantly affected W_max_; the interaction was not significant for any grain-filling parameter. Across years, density and irrigation affected Vmax, Wmax, and D. PD2 increased Vmax by 2.5%–7.8% over PD1 and PD3, while I1 elevated Vmax, Wmax, and D by 9.3%, 12.5%, and 2.0%, respectively, over I2 and I3. The highest Wmax was found under PD1I1 and PD1I2, exhibiting a 28.2%–34.7% increase over PD3I3, along with a 7.1%–18.5% extension of the active filling period.

**Table 5:** The grain-filling process model and grain-filling parameters under different irrigation treatments.

Year	Treatment	Growth curve equation	Correlation coefficient	T_max_	W_max_	V_max_	V_mean_	D
2024	PD1I1	y=40.3/(1 + 52.19e^-0.2254x^)	0.9918	17.6 a	20.2 a	2.3 ab	1.1 abc	26.4 abc
	PD1I2	y=37.98/(1 + 38.92e^-0.216x^)	0.9929	16.9 a	19.0 ab	2.1 bcd	1.0 bc	27.7 ab
	PD1I3	y=31.75/(1 + 40.82e^-0.2459x^)	0.9941	15.1 bc	15.9 cd	2.0 bcd	0.9 c	24.4 bc
	PD2I1	y=40.41/(1 + 68.39e^-0.2748x^)	0.9949	17.1 a	20.2 a	2.5 a	1.1 ab	24.1 bc
	PD2I2	y=36.81/(1 + 25.83e^-0.2098x^)	0.9890	15.6 b	18.5 ab	1.9 bcd	1.0 bc	28.9 ab
	PD2I3	y=32.28/(1 + 17.64e^-0.2093x^)	0.9908	13.8 d	16.2 cd	1.7 d	0.9 c	28.6 ab
	PD3I1	y=39.43/(1 + 17.68e^-0.2238x^)	0.9871	12.8 d	19.7 a	2.2 abc	1.2 a	26.8 abc
	PD3I2	y=35.15/(1 + 14.71e^-0.2068x^)	0.9516	13.1 d	17.6 bc	1.8 cd	1.0 bc	29.5 a
	PD3I3	y=29.92/(1 + 39.39e^-0.264x^)	0.9504	13.9 cd	15.0 d	2.0 bcd	1.0 bc	22.5 c
2025	PD1I1	y=41.96/(1 + 31.18e^-0.1962x^)	0.9917	17.7 b	21.1 a	2.1 ab	1.0 a	30.7 abc
	PD1I2	y=42.17/(1 + 29.49e^-0.1732x^)	0.9953	19.5 a	21.1 a	1.8 b	0.9 b	34.7 a
	PD1I3	y=38.46/(1 + 28.72e^-0.1831x^)	0.9844	18.4a b	19.3 cd	1.8 b	0.9 b	32.5 ab
	PD2I1	y=41.8/(1 + 30.55e^-0.1871x^)	0.9907	18.3 ab	20.9 ab	2.0 ab	1.0 ab	32.0 ab
	PD2I2	y=40.92/(1 + 31.32e^-0.2156x^)	0.9935	16.0 cd	20.5 ab	2.2 a	1.1 a	27.9 bc
	PD2I3	y=37.47/(1 + 35.92e^-0.2324x^)	0.9960	15.4 d	18.7 d	2.2 a	1.1 a	25.7 c
	PD3I1	y=40.06/(1 + 27.17e^-0.1725x^)	0.9922	19.1 ab	20.0 bc	1.7 b	0.9 b	34.6 a
	PD3I2	y=39.89/(1 + 30.81e^-0.1761x^)	0.9917	19.5 a	20.0 bc	1.8 b	0.9 b	34.3 a
	PD3I3	y=32.58/(1 + 48.48e^-0.2219x^)	0.9912	17.5 bc	16.3 e	1.8 b	0.9 a	27.0 bc
Planting density (PD)				ns	*	ns	ns	ns
Irrigation (I)				ns	**	*	*	*
PD × I				ns	ns	ns	ns	ns

Tmax, the time required to reach maximum grain–filling rate; T, the duration of grain filling; Wmax, the grain weight at maximum grain filling rate; Vmax, maximum grain filling rate; Vmean, mean grain filling rate; D, active grain filling period. Values followed by the same lowercase letter within a column for a given year are not significantly different at P < 0.05 according to the LSD test. * and ** indicate significant effects at the 0.05 and 0.01 probability levels, respectively; ns indicates not significant.

### 3.7 Hormonal status and oxidative stress related traits

**Figure 8:**
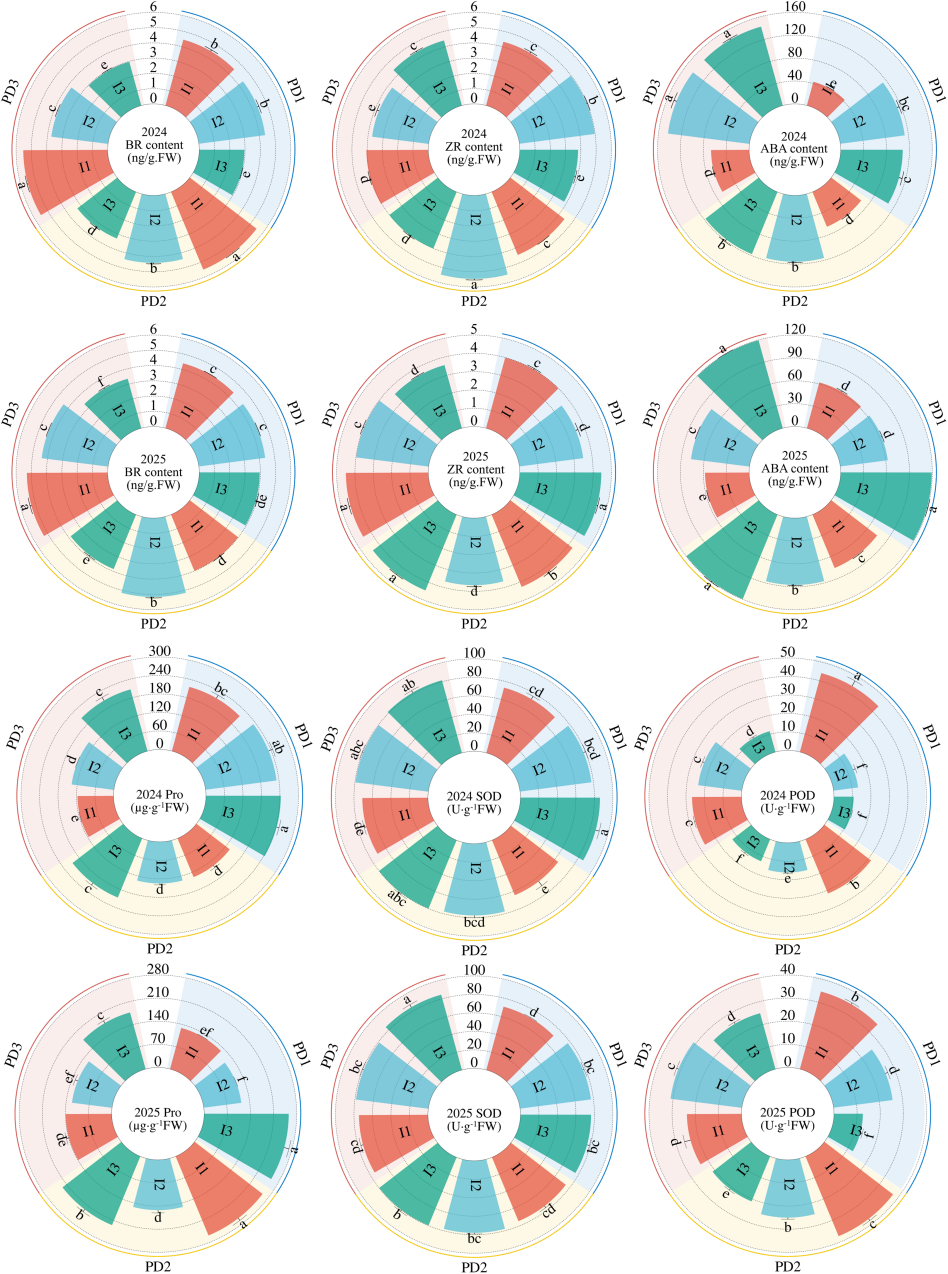
Effects of irrigation and planting density on endogenous hormones (BR, ZR, and ABA) in wheat in 2024 and 2025. The data are shown as the means ± SDs. Different letters indicate significant differences between different treatments (*P* < 0.05).

During grain filling, BR, ZR, and ABA were significantly influenced by PD, I, and their interaction (P < 0.001; **Figure 8**). Across years, PD2 and PD3 increased BR by 6.9% and 2.9% relative to PD1, respectively. Irrigation exerted a greater impact on hormone levels than density did. In 2024, BR under I1 was 23.7% and 68.4% higher than under I2 and I3. ZR peaked under I2 in 2024, with values 10.3% and 15.7% above those of I1 and I3, respectively, whereas in 2025 the peak occurred under I1. Reduced irrigation promoted ABA accumulation: in 2024, I2 and I3 raised ABA by 112.8% and 110.4% over I1; in 2025, I3 exceeded I1 and I2 by 83.2% and 57.5%.

Furthermore, irrigation significantly affected PRO, POD, and SOD (P < 0.001; **Figure 8**). In 2024, PRO under I2 and I3 increased by 7.7% and 40.3% over I1; SOD rose by 12.6% and 18.7% in 2024 and by 6.2% and 9.2% in 2025, respectively. Density effects were comparatively weaker. Overall, reduced irrigation induced ABA accumulation and enhanced osmotic and antioxidant responses during grain filling.

### 3.8 Correlation analysis and structural equation modeling

**Figure 9:**
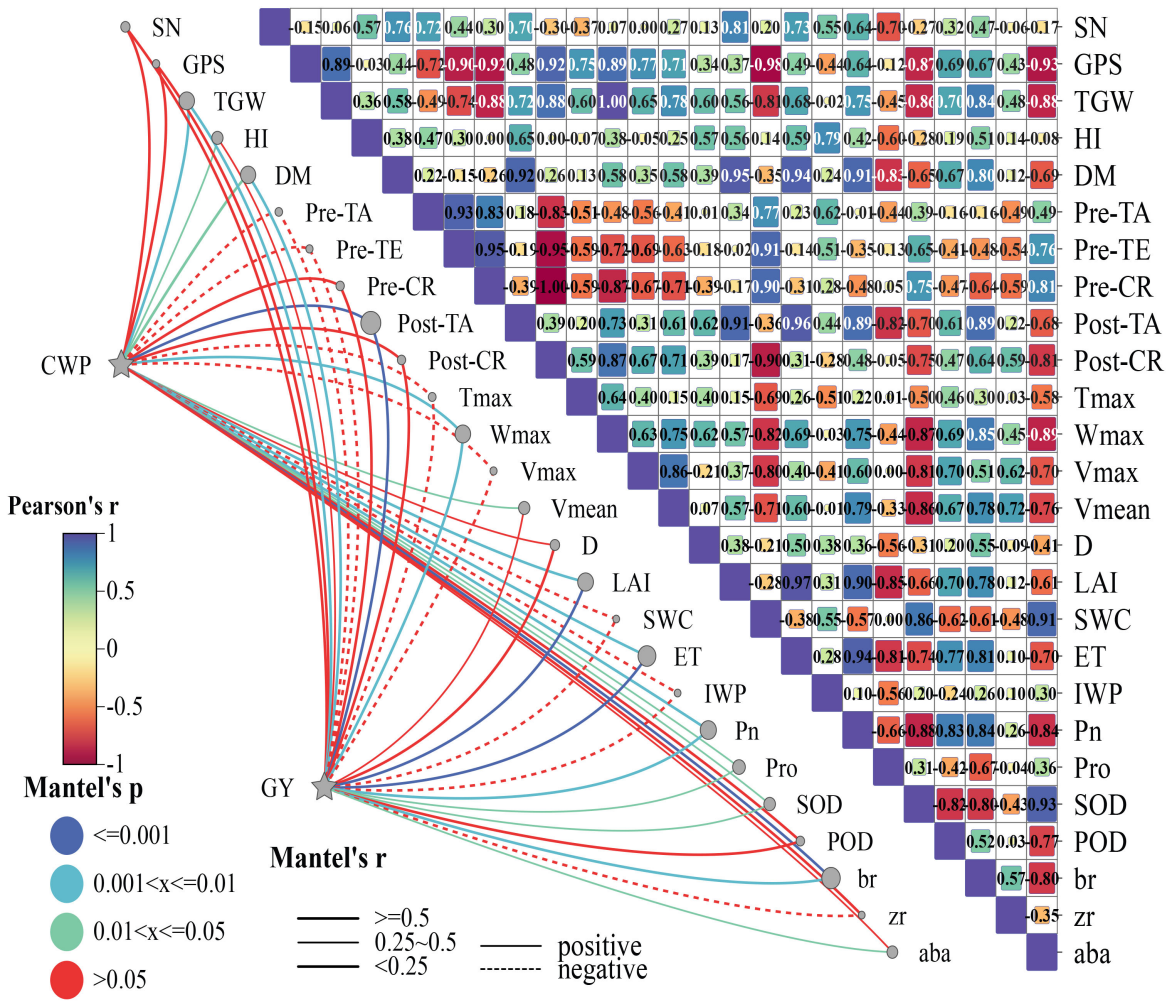
The heat map of the correlation analysis and Mantel analysis between crop water productivity and grain yield and various indicators.

Correlation analysis showed that WP_C_ and GY were positively associated with TA_Pre_, CR_Pre_, Vmax, and D, but negatively associated with LAI and ET, while Post-TA and Post-CR were not significantly related to SWC (**Figure 9**). Mantel tests further indicated that both WP_C_ and GY were positively correlated with TGW, HI, DM, WP_I_, and Pn, and negatively correlated with LAI, ET, and ABA. In addition, Vmean was positively related to GY, and TGW was positively related to WP_C_, whereas SOD and POD showed no significant relationships with GY.

**Figure 10:**
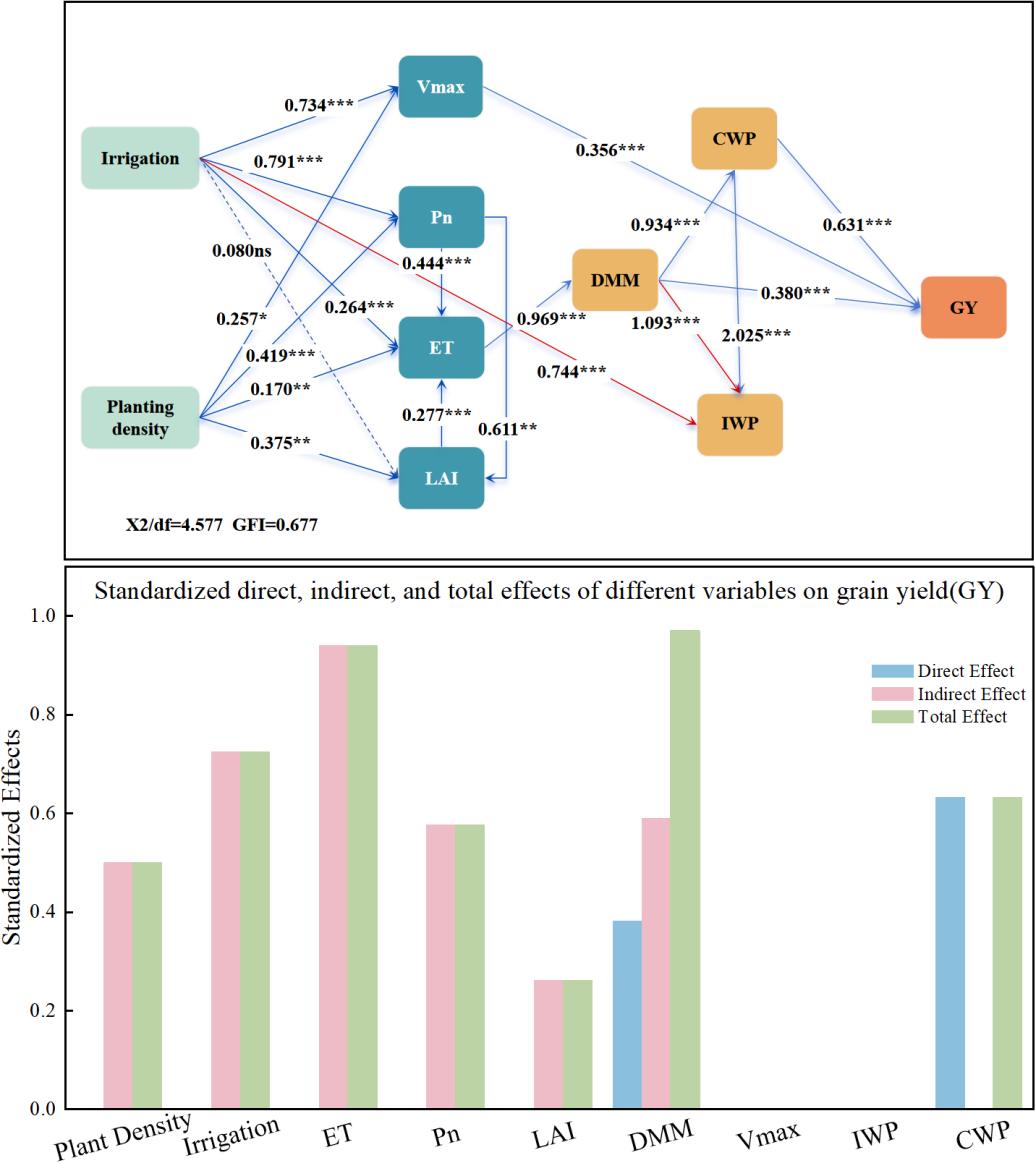
Structural equation model illustrating the pathways by which irrigation level and planting density regulate grain yield and water productivity in spring wheat. * P < 0.05, ** P < 0.01, *** P < 0.001.

Structural equation modeling showed acceptable fit (χ^2^/df = 4.577, GFI = 0.829). Among the nine variables influencing GY, DMM (Dry matter accumulation at maturity, 0.97), ET (0.939), and Irrigation (0.724) exhibited the highest total effects, while Plant Density (0.499) and Pn (0.576) were relatively lower. Path analysis indicated that Irrigation (0.724) and ET (0.939) acted entirely through indirect pathways (direct effects = 0), whereas DMM and CWP showed strong positive direct effects (0.38 and 0.631, respectively), with DMM also having considerable indirect effects (0.589). Collectively, these results suggest that DMM and CWP were the primary direct drivers of GY, while Irrigation and ET regulated yield mainly via indirect pathways (**Figure 10**).

## 4 Discussion

### 4.1 Yield–water productivity trade-off under irrigation–density coupling

The present study demonstrated that the treatment maximizing grain yield was not identical to that optimizing water productivity, reflecting an inherent trade-off between production potential and water-use efficiency under shallow-buried drip irrigation (Yang et al., 2025). Across both years, PD3I1 achieved the highest grain yield, whereas PD2I2 consistently resulted in superior WP_C_ and WP_I_ (**Tables 2**, **4**). This divergence indicates that yield and water productivity respond differently to irrigation and density, and are optimized through separate management adjustments (Dhakar et al., 2023; Liu et al., 2024). High irrigation combined with high density enhanced sink capacity at the population level, mainly through increased spike number, but this came at the cost of reduced resource allocation per spike and lower water-use efficiency. In contrast, the PD2I2 combination appeared to optimize the balance between canopy water demand and soil water supply, thereby improving the efficiency of carbon assimilation and its conversion into grain yield.

From an agronomic perspective, these results highlight the need to coordinate source activity, sink capacity, and water availability through integrated irrigation and density management (Fang et al., 2024). Previous studies have shown that moderate deficit irrigation can improve water productivity without substantial yield penalties by enhancing water-use efficiency at both canopy and field scales (Yang et al., 2025; Yu et al., 2020). Similarly, optimized planting density can regulate canopy structure and improve radiation interception and resource distribution within the population (Dai et al., 2022; Gao et al., 2021). However, the present study demonstrates that under shallow-buried drip irrigation, the optimal management strategy depends on the synchronization between spatial water supply (controlled by drip irrigation patterns) and population-level water demand (regulated by planting density). When density exceeds the capacity of the localized wetting zone to supply water, competition intensifies and reduces the marginal benefit of additional plants, even under sufficient irrigation.

### 4.2 Soil water redistribution and evapotranspiration reveal the supply-side constraint

A key implication of this study is that total seasonal ET alone was insufficient to explain treatment differences, because ET varied within a relatively narrow range while ΔSWS, WP_C_, and WP_I_ differed significantly. This suggests that water productivity under shallow-buried drip irrigation is controlled less by total water consumption than by when and where water is extracted (Jha et al., 2017). Moderate irrigation under PD2 likely improved the temporal match between soil water availability and reproductive demand, whereas high density under low irrigation accelerated profile depletion and weakened the yield benefit of additional plants. This interpretation is consistent with recent evidence that wheat yield and water productivity depend on coordinated below- and above-ground growth, rather than on water input alone (Fu et al., 2025). Therefore, the present results extend conventional deficit-irrigation theory by emphasizing the spatial coupling between the drip-induced wetting pattern and crop population demand (Yang et al., 2025; Yu et al., 2020).

### 4.3 Source–sink regulation and physiological responses explain the demand-side limitation

The contrasting responses of yield and water productivity indicate that crop performance under shallow-buried drip irrigation is primarily constrained by demand-side regulation rather than water supply alone. In this study, PD3I1 maximized grain yield, whereas PD2I2 achieved higher water productivity, reflecting a decoupling between population sink size and sink realization. Increasing planting density enlarged population sink capacity, but under limited water supply, individual spike filling was restricted, leading to reduced grain weight (Liu et al., 2024). Similar patterns have been reported in recent studies, where excessive canopy development intensified intra-population competition and limited grain filling despite favorable resource conditions (Fang et al., 2024; Liu et al., 2024). Although deficit irrigation enhanced pre-anthesis remobilization, grain filling remained predominantly dependent on post-anthesis assimilation, and increased translocation could not fully compensate for reduced photosynthetic supply (Fang et al., 2024; Moradi et al., 2022). These results suggest that optimizing yield and water productivity requires balancing sink size with assimilate supply rather than maximizing either component independently.

Physiological responses further confirm this demand-side limitation. In this study, PD2I2 maintained higher Pn, Tr, and Gs around anthesis, supporting sustained carbon assimilation, whereas higher ABA, PRO, and antioxidant activity under I3 indicated stress adaptation rather than productive gain. Consistent evidence shows that maintaining post-anthesis photosynthesis is more critical for yield stability than enhancing stress-related responses under water deficit (Dai et al., 2022; Ru et al., 2024). Elevated ABA and antioxidant activity typically reflect a shift toward survival-oriented metabolism at the expense of carbon assimilation (Luo et al., 2021). Therefore, the advantage of PD2I2 can be attributed to improved synchronization between canopy demand and resource supply, sustaining both photosynthetic capacity and grain filling. This study applies the source-sink framework to evaluate practical irrigation–density combinations, demonstrating that water availability acts as a key limiting factor and that management should balance source and sink capacities. These findings provide practical guidance for optimizing yield and water productivity under localized irrigation systems (Liu et al., 2024).

### 4.4 Limitations and future directions

This study is limited to two seasons, one site, and one cultivar under a specific shallow buried drip configuration, which constrains direct extrapolation to other oasis districts, soils, cultivars, and irrigation designs. Accordingly, while PD2I2 emerged as a promising water−saving practice in this study, its broader applicability across different climatic regimes, soil types, wheat cultivars, alternative drip configurations, and planting density ranges remains to be tested. The present results should therefore be viewed as providing a proof−of−concept under representative oasis conditions, and the boundary conditions of this recommendation warrant systematic evaluation in future multi−site, multi−cultivar experiments. Root distribution, soil evaporation, and transpiration were not directly quantified, so the mechanistic attribution of water productivity differences to ET component shifts remains inferential, even though it is consistent with prior drip irrigation evidence (Yang et al., 2020a). In addition, the hormone and stress indicators were measured at a single grain filling stage, which limits interpretation of causal timing relative to changes in photosynthesis, assimilate remobilization, and grain filling dynamics.

Future work should validate the density and irrigation recommendations across more years, multiple cultivars with contrasting canopy and root traits, and different drip design parameters, while directly partitioning evapotranspiration and measuring root length density across soil depths. Repeated time series sampling of hormones and antioxidant traits across grain filling would also clarify whether hormonal shifts precede or follow changes in photosynthesis and assimilate allocation, strengthening process-based inference and supporting more robust management decision rules.

## 5 Conclusion

This study demonstrated that irrigation level and planting density jointly regulate grain yield and water productivity of spring wheat under shallow-buried drip irrigation, but their optimal combinations differ depending on the management objective. High irrigation combined with high density (PD3I1) maximized grain yield, whereas moderate irrigation and density (PD2I2) achieved superior water productivity and is recommended for long-term water saving and stable yield in the water-scarce Hexi Oasis Irrigation Area. Grain filling and physiological responses further showed that under limited water supply, the increased sink capacity under high density could not be fully realized, highlighting the importance of matching sink size with assimilate supply through coordinated irrigation and density. Moderate density and irrigation maintained a better balance between canopy development, photosynthetic activity, and grain filling, resulting in improved coordination between source activity and sink realization. Overall, the results highlight that optimizing wheat production under water-limited conditions requires synchronizing population structure with irrigation-induced soil water availability, rather than maximizing irrigation or planting density alone. This provides a practical basis for water-saving and high-efficiency management of wheat in the Hexi arid oasis irrigation systems.

## Data Availability

The original contributions presented in the study are included in the article/supplementary material. Further inquiries can be directed to the corresponding authors.
